# Predictive modeling of hemoglobin refractive index using Gaussian process regression with interpretability through partial dependence plots

**DOI:** 10.1371/journal.pone.0324827

**Published:** 2025-05-30

**Authors:** Mustfa Faisal Alkhanani

**Affiliations:** Biology Department, College of Science, University of Hafr Al Batin, Hafr Al Batin, Saudi Arabia; Universite Cote d'Azur, FRANCE

## Abstract

Accurately predicting the refractive index of hemoglobin across various wavelengths and concentrations is critical for advancing optical diagnostic techniques in biological and clinical applications. This study introduces a predictive model based on Gaussian Process Regression (GPR) to estimate the refractive index of hemoglobin in both oxygenated and deoxygenated states, covering wavelengths from 400 to 700 nm and concentrations ranging from 0 to 140 g/L. The GPR model effectively captures non-linear relationships, achieving high prediction accuracy with R^2^ values of 99.4% for the training dataset and 99.3% for the testing dataset. An independent external dataset was used to validate the model’s robustness further, yielding an R^2^ value of 92.80%, RMSE of 0.0042, and MSE of 1.77 × 10 ⁻ ⁵, demonstrating the model’s strong generalizability. To enhance interpretability, Partial Dependence Plots (PDPs) were employed to visualize the influence of wavelength and concentration on refractive index predictions, offering clear insights into hemoglobin’s optical behavior. The model’s ability to provide accurate and interpretable predictions has significant implications for improving the reliability of biophotonic diagnostic tools, such as optical coherence tomography and reflectance spectroscopy, in clinical settings. By combining machine learning with interpretability techniques, this study advances the understanding of hemoglobin’s optical properties and sets a benchmark for predictive modeling in biomedical optics, paving the way for more precise and dependable diagnostic applications.

## 1.0 Introduction

Hemoglobin is the oxygen-carrying protein in red blood cells essential for various physiological processes [1,2]. Its concentration, structural conformation, and interaction with light are crucial for medical diagnostics, particularly in monitoring conditions such as anemia, hemolysis, and other blood disorders [3–5]. The optical properties of hemoglobin and its interaction with diagnostic tools like optical coherence tomography (OCT), reflectance spectroscopy, and other photonic technologies largely depend on its refractive index (RI) [6–9]. The refractive index of hemoglobin is influenced by several factors, including the wavelength of incident light, its concentration, and its oxygenation state [10,11]. Historically, directly measuring hemoglobin’s refractive index across different concentrations and wavelengths has been challenging, despite its importance [12,13]. In earlier studies, indirect methods were primarily used to estimate the refractive index. While these methods provided valuable insights, they often led to inconsistent results [12,14]. As a result, there is a growing need for accurate and robust predictive models that can reliably estimate hemoglobin’s refractive index under various physiological conditions. This study addresses this need by employing machine learning techniques, specifically Gaussian Process Regression (GPR), to predict hemoglobin’s refractive index.

Research on hemoglobin’s refractive index has a long history. Early studies by Barer (1957) and other researchers in the mid-20th century focused on quantifying hemoglobin’s refractive index using limited wavelength data, particularly at 589 nm [13]. Barer’s work established a fundamental linear correlation between hemoglobin concentration and refractive index, which has been widely referenced in the field [15]. However, as biophotonics advanced, researchers recognized the limitations of these early models, especially their inability to account for the complexities introduced by varying wavelengths and hemoglobin states such as oxygenated and deoxygenated hemoglobin [16–18]. In recent decades, advancements in optical measurement techniques have enabled researchers to explore hemoglobin’s refractive index across a broader range of wavelengths [8,12,19,20]. For example, Friebel and Meinke used indirect methods, such as the Kramers–Kronig relations and simultaneous reflection and absorption measurements, to estimate hemoglobin’s refractive index over an extended spectral range [10,21–24]. Despite these advancements, inconsistencies in experimental conditions—such as differences in hemoglobin extraction methods and measurement protocols—often led to discrepancies in findings across research groups [24].

Zhernovaya et al. made significant contributions by directly measuring the refractive index of oxygenated and deoxygenated hemoglobin at nine wavelengths ranging from 400 to 700 nm [15]. Their findings confirmed the linear relationship between hemoglobin concentration and refractive index while identifying previously unknown regions of anomalous dispersion. These results provided a deeper understanding of hemoglobin’s optical properties, but the challenge of developing a broadly applicable predictive model remained unresolved. At the same time, advancements in hemoglobin research have been accompanied by significant progress in machine learning. Predictive models, particularly those based on Gaussian Process Regression (GPR), have become increasingly popular due to their ability to capture complex, non-linear relationships [25]. GPR, a non-parametric Bayesian technique, not only provides predictions but also estimates uncertainty, making it a powerful tool for modeling biological phenomena with inherent variability [26–28]. However, a common criticism of GPR and other machine learning models is their black box nature, which makes it difficult to interpret how specific features influence the predictions [29,30]. To address this limitation, techniques such as partial dependence plots (PDPs) have been developed [31,32]. PDPs visually illustrate how individual features, such as hemoglobin concentration and wavelength, impact the model’s predictions, bridging the gap between the accuracy of machine learning models and the interpretability required in scientific research [29,33]. Combining GPR with PDPs makes achieving both high predictive accuracy and a clearer understanding of the underlying physical phenomena possible.

This study builds on previous work by employing a machine learning approach to predict the refractive index of hemoglobin, leveraging the strengths of GPR and PDPs to enhance both accuracy and interpretability. The main contribution of this study is the development of a Gaussian Process Regression (GPR) model to predict the refractive index of hemoglobin across different wavelengths. The GPR model was chosen for its ability to capture complex, non-linear relationships. Unlike previous studies focusing on a narrow range of wavelengths or specific hemoglobin states, such as oxygenated or deoxygenated, this work integrates data across a broad spectral range (400–700 nm) and both oxygenation states. This comprehensive approach allows the GPR model to fully represent hemoglobin’s optical complexity, resulting in more robust and generalizable predictions. Additionally, while GPR provides reliable predictions, this study goes further by using Partial Dependence Plots (PDPs) to interpret how input parameters (e.g., hemoglobin concentration, wavelength) influence the refractive index. This interpretability enhances the model’s transparency and provides valuable insights into hemoglobin’s optical behavior, which is critical for clinical and diagnostic applications.

The remainder of the study is organized as follows: The materials and methods section describes the dataset, modeling techniques, optimization methods, and PDPs. The result section evaluates the GPR model’s predictive accuracy and interprets the results using PDPs. The discussion section discusses the findings in the context of current knowledge and explores their implications for future research and clinical applications. Finally, the conclusion section summarizes the key findings and suggests directions for future investigation.

## 2 Materials and methods

### 2.1 Data collection

The data used for this study were sourced from Zhernovaya et al., who measured the refractive index of human hemoglobin in both oxygenated and deoxygenated states across nine wavelengths in the visible range (400–700 nm) [15]. The refractive index was determined using a digital multiwavelength refractometer (DSR-λ, Schmidt & Haensch™, Germany) based on total internal reflection. The training dataset covers hemoglobin concentrations from 0 to 140 g/L, which includes physiological extremes such as severe anemia (<50 g/L) and normal levels (120–160 g/L for adults), as well as experimental conditions relevant to in vitro diagnostics. This range ensures the model applies to both clinical and laboratory settings. Temperature control was maintained at 20 °C to minimize evaporation, and additional measurements were taken at 37 °C to assess thermal effects. These measurements provide a foundational dataset for a predictive hemoglobin refractive index model.

**Fig 1:**
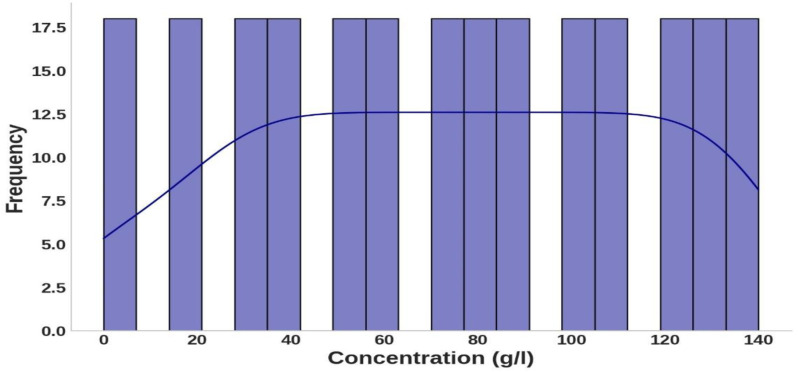
The distribution of hemoglobin concentration values across the dataset illustrates the frequency and range of hemoglobin concentrations measured with a Kernel Density Estimate line.

The descriptive statistics in Table 1 highlight the dataset’s central tendencies and variability. The mean, median, and standard deviations for hemoglobin concentration, wavelength, and refractive index illustrate the data’s diversity, confirming its suitability for Gaussian Process Regression (GPR). The dataset’s range in both concentration and wavelength aids in capturing complex, non-linear relationships, which is essential for studying optical interactions with biological tissues in the visible spectrum. The distribution analysis reveals notable trends within the dataset. The refractive index was measured at multiple concentrations, and its values were found to vary linearly with hemoglobin concentration. The hemoglobin concentration distribution in Fig 1 displays a moderate spread with clusters around specific concentrations, which may affect model generalizability. For infrequent concentration values, the model may rely more heavily on interpolation.

**Table 1:** Statistics of the dataset for hemoglobin concentration, wavelength, and refractive index variables [15].

Statistics	Concentration (g/L)	Wavelength (nm)	Refractive Index
Count	252.000	252.000	252.000
Mean	74.286	560.222	1.349
Std	41.610	96.720	0.008
Min	0.000	401.500	1.332
Max	140.000	706.500	1.369

**Fig 2:**
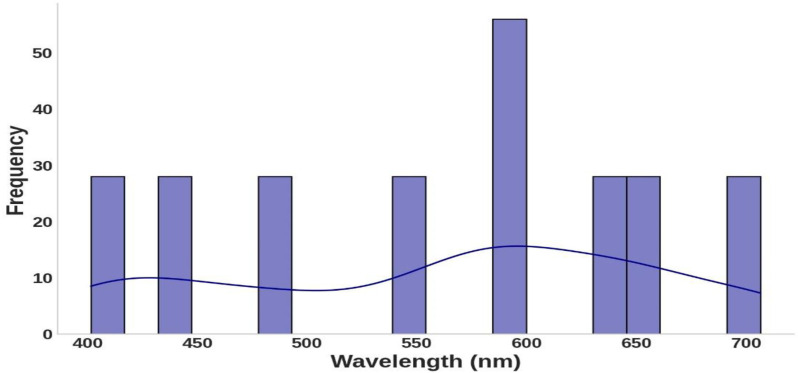
The distribution of wavelength values in the visible spectrum (400–700 nm) in the dataset highlights the wavelengths used for refractive index measurements and analysis.

**Fig 3:**
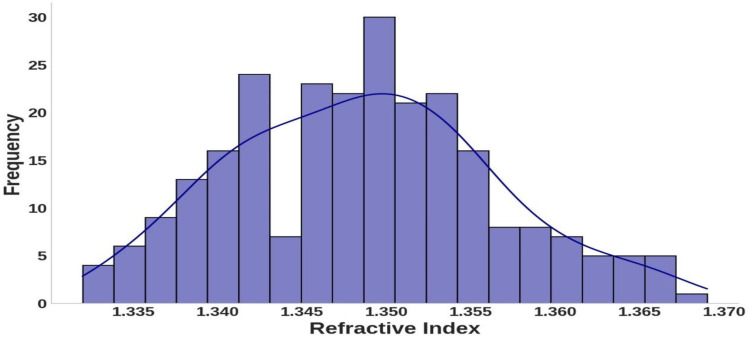
Distribution of refractive index values observed within the dataset.

Similarly, Fig 2 shows a relatively even spread of wavelengths across the visible spectrum, which is significant for photonics and biomedicine. This distribution supports examining hemoglobin’s interaction with light in clinically relevant ranges. The refractive index distribution, depicted in Fig 3, demonstrates consistent responsiveness to hemoglobin concentration and wavelength variations. The histogram and overlaid curve reveal that refractive index values are concentrated around a central value of approximately 1.350, with frequencies peaking in this range. This suggests that under experimental conditions, hemoglobin’s refractive index stabilizes near this value. The symmetric shape of the distribution indicates that variations in concentration and wavelength cause relatively uniform shifts around the mean refractive index, with no extreme deviations. This consistency highlights the predictable optical properties of hemoglobin across the tested conditions.

The moderate spread of the data reflects some variation in refractive index, likely due to changes in hemoglobin concentration or wavelength. However, these variations remain within a defined range, underscoring the material’s stable interaction with light. These insights are critical for understanding hemoglobin’s optical characteristics, as the refractive index is vital in applications such as diagnostic imaging and spectroscopy. The observed patterns suggest that the interaction between light and hemoglobin is governed by regular, quantifiable changes, which can inform the development of precise analytical models or medical devices for monitoring hemoglobin properties.

**Fig 4:**
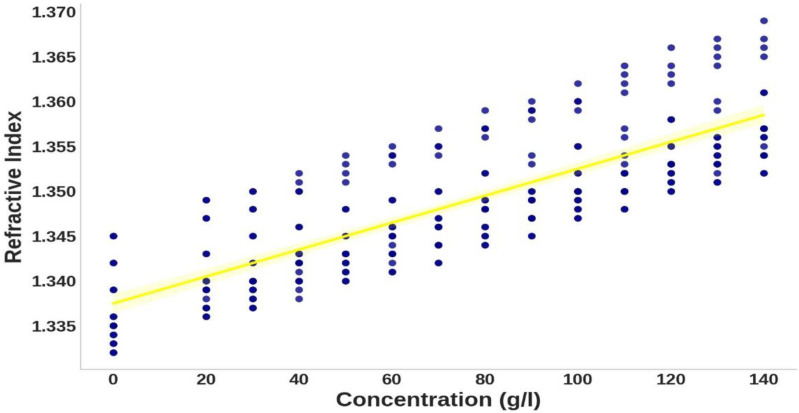
Scatter plot of hemoglobin concentration versus refractive index.

**Fig 5:**
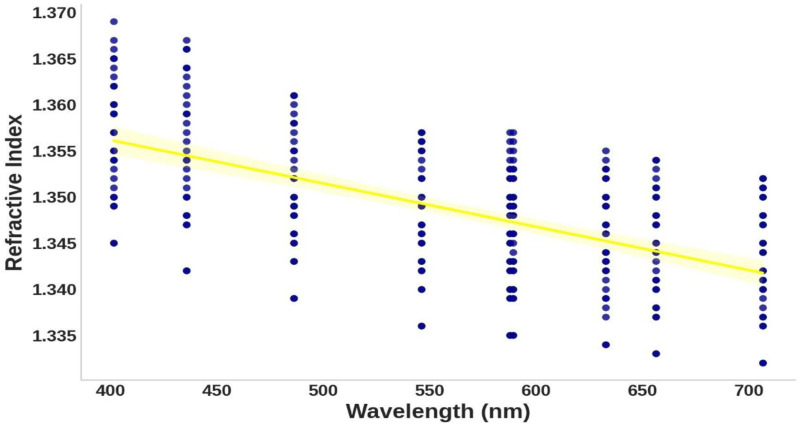
Scatter plot of wavelength versus refractive index.

**Fig 6:**
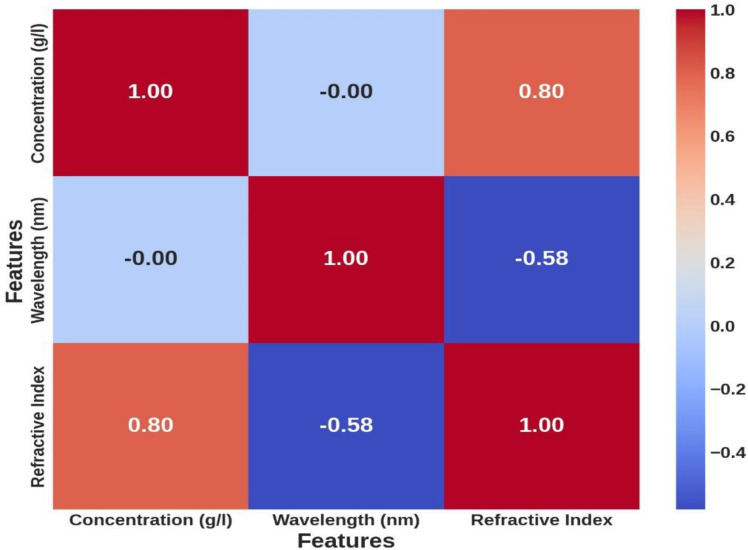
Correlation matrix depicting the relationships between hemoglobin concentration, wavelength, and refractive index.

Analyzing the relationship between concentration and refractive index in Fig 4 reveals a positive correlation, where higher hemoglobin concentrations correspond to elevated refractive index values. This trend aligns with optical principles, as denser media have a more significant impact on light propagation, highlighting the importance of hemoglobin concentration in predictive modeling [12]. Conversely, Fig 5 shows an inverse relationship between wavelength and refractive index, with the refractive index decreasing as wavelength increases. This behavior is consistent with optical dispersion principles and emphasizes the complex interplay between concentration, wavelength, and refractive index [12]. These observations demonstrate the need for a non-linear model like GPR to capture these dependencies accurately. Finally, the correlation matrix in Fig 6 quantifies the relationships between concentration, wavelength, and refractive index, showing high correlation coefficients. These correlations validate the selection of concentration and wavelength as model inputs and provide a strong foundation for the Gaussian Process Regression (GPR).

#### 2.1.1 External data validation.

An independent external dataset was used to validate the robustness and generalizability of the Gaussian Process Regression (GPR) model. This dataset, obtained from Yahya and Saghir [34], includes 35 observations with hemoglobin concentrations ranging from 0 to 260 g/L and wavelengths spanning 480–680 nm. The refractive index values in this dataset range from 1.3301 to 1.3879, extending into supraphysiological conditions (e.g., hemoconcentration studies), which are relevant for laboratory settings. Key performance metrics, including R^2^, RMSE, and MSE, were used to evaluate the model’s predictive accuracy on this external dataset.

### 2.2 Gaussian Process Regression (GPR) model

Gaussian Process Regression (GPR) is a non-parametric, Bayesian regression method that predicts data by positing that observations are sampled from a Gaussian process, a set of random variables exhibiting a joint Gaussian distribution [8]. Unlike traditional regression models, Gaussian Process Regression (GPR) does not presuppose a particular functional form between inputs and outputs, rendering it exceptionally adaptable and proficient at identifying intricate, non-linear associations inside the data [26,27,35] The adaptability and interpretability of Gaussian Process Regression make it an optimal selection for modelling the refractive index of hemoglobin, which demonstrates non-linear dependencies on wavelength.

### 2.3 Bayesian optimization

**Fig 7:**
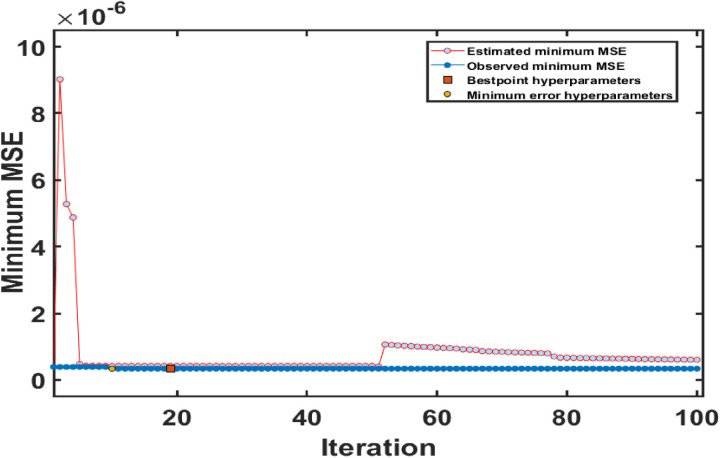
Optimization plot showing Minimum Mean Squared Error (MSE) across GPR model iteration.

Bayesian optimization is an effective method for tuning hyperparameters in Gaussian Process Regression (GPR) models, aimed at enhancing predictive performance and minimizing error metrics for accurately estimating hemoglobin’s refractive index [36–38]. In this study, the key hyperparameters were fine-tuned and the results are summarized in Table 2. The isotropic exponential kernel function was chosen due to model covariance, ensuring uniform behaviour across predictors - hemoglobin concentration and wavelength. The kernel scale was set to 12.1943, allowing the model to capture complex relationships without being overly sensitive to noise. A sigma value of 0.031299 indicated a reliable noise level, essential for accurate optical measurements. Additionally, standardizing the data ensured that each feature contributed equally to the model, preventing disproportionate influence. The optimization process is illustrated in Fig 7, which shows the convergence of the model, with the Minimum Mean Squared Error (MSE) achieved within the first 20 iterations. This approach streamlined hyperparameter tuning and ensured that the GPR model remained efficient and robust, underscoring its effectiveness in modelling complex biological phenomena like hemoglobin’s refractive index.

**Table 2:** Optimized hyperparameters for the Gaussian Process Regression (GPR) model.

Hyperparameter	Optimized Value
Basis Function	Linear
Kernel Function	Isotropic Exponential
Kernel Scale	12.1943
Sigma	0.031299
Standardize Data	Yes

### 2.4 Computational procedures

#### 2.4.1 Model training and validation.

The GPR model was implemented in MATLAB 2024b software. Hyperparameters were fine-tuned using Bayesian optimization, a sequential model-based approach that balances exploration and exploitation of the hyperparameter space to minimize the Root Mean Square Error (RMSE) [39]. This method constructs a probabilistic surrogate objective function model, ensuring efficient and robust optimization. To enhance generalizability, a 5-fold cross-validation strategy was employed. The dataset was split into five subsets, with each subset serving as the validation set once while the remaining four were used for training. This process was repeated five times, ensuring all data points were used for both training and validation [40,41]. The final model was trained on 80% of the data and tested on the remaining 20%, minimizing overfitting and maximizing prediction accuracy.

**Fig 8:**
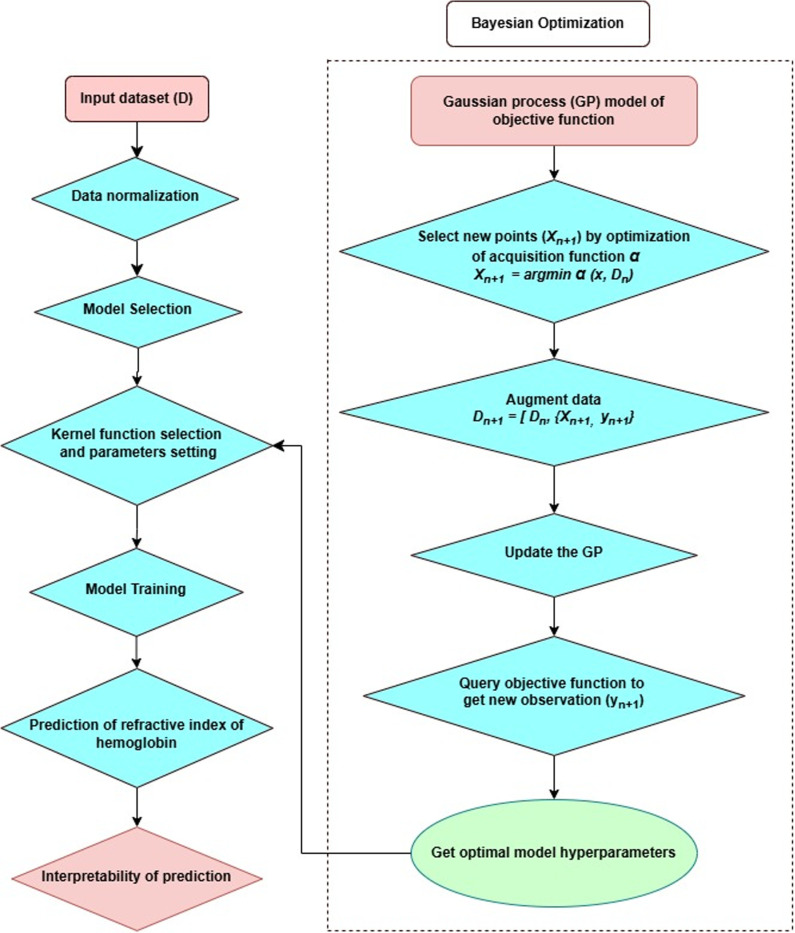
The flowchart outlines the key steps, including data collection, preprocessing, model training, validation, optimization and interpretation.

Model performance was evaluated using key metrics: R^2^, RMSE, and Mean Squared Error (MSE). The GPR model achieved an R^2^ of 99.4% for the training set and 99.3% for the testing set, with RMSE values of 0.00061 and 0.00062, respectively. These results indicate a strong fit between predicted and actual refractive index values, with minimal prediction errors, demonstrating the model’s robustness for applications in biophotonic diagnostics. The study workflow, illustrated in Fig 8 outlines the key steps from data preprocessing to model evaluation. Preprocessing steps, including normalization and feature selection, followed data collection. The GPR model was trained using Bayesian optimization, and 5-fold cross-validation was applied to enhance generalizability. Partial Dependence Plots (PDPs) were generated to interpret the influence of input features on refractive index predictions.

#### 2.4.2 Performance metrics.

The generalization performance of the developed models was evaluated using mean squared error (MSE) and root mean square error (RMSE). The equations used are presented in equations 1–3.

Mean Squared Error (MSE): The MSE measures the average squared difference between actual and predicted values, with lower values indicating better model accuracy. It can be computed using equation 1.


$$MSE=\sum_{i=0}^{n}{\left(y_{i\;}-\;{\hat{y}}_{i\;}\right)}^{2}$$ (1)

where n = Number of observations, $y_{i\;}$= Actual value for the i-th observation, ${\hat{y}}_{i\;}$ = Predicted value for the i-th observation.

**Root Mean Squared Error (RMSE):** The RMSE is the square root of the MSE and measures the average magnitude of the prediction errors in the same units as the target variable. It is calculated as shown in equation 2.


$$RMSE=\sqrt{\sum_{i=0}^{n}{\left(y_{i\;}-\;{\hat{y}}_{i\;}\right)}^{2}}$$ (2)

where n = Number of observations, $y_{i\;}$= Actual value for the i-th observation, ${\hat{y}}_{i\;}$ = Predicted value for the i-th observation.

**R**^2^ **(Coefficient of Determination):**

R^2^ measures the proportion of variance in the dependent variable that is predictable from the independent variables. It ranges from 0 to 1, with higher values indicating a better fit. It is calculated as shown in equation 3.


$$R^{2}=1-\frac{\sum_{i=0}^{n}{\left(y_{i\;}-\;{\hat{y}}_{i\;}\right)}^{2}}{\sum_{i=0}^{n}{\left(y_{i\;}-\;y\;\right)}^{2}}$$ (3)

where n = Number of observations, $y_{i\;}$= Actual value for the i-th observation, ${\hat{y}}_{i\;}$ = Predicted value for the i-th observation and y  = mean of the actual values.

#### 2.4.2 Partial Dependence Plot (PDP) Analysis.

Partial Dependence Plot (PDP) analysis was performed in MATLAB to improve the interpretability of the Gaussian Process Regression (GPR) model. This analysis visualized the influence of individual input features—hemoglobin concentration and wavelength—on the predicted refractive index. PDPs are particularly valuable in biomedical applications, as they isolate the effect of each feature, providing critical insights into the model’s behavior and enhancing transparency [42].

For this study, PDPs were generated by holding one input variable constant at its mean value while varying the other. In the case of hemoglobin concentration, the wavelength was fixed at its mean, allowing the PDP to demonstrate how changes in concentration alone affect refractive index predictions. Conversely, for the wavelength PDP, hemoglobin concentration was held constant at its mean while the wavelength was varied. This analysis revealed an inverse relationship between wavelength and refractive index. These PDPs clearly understand how each feature contributes to the model’s predictions, highlighting the non-linear relationships captured by the GPR model. By combining predictive accuracy with interpretability, the PDPs provide valuable insights into the optical properties of hemoglobin, which are essential for advancing biophotonic diagnostic techniques.

## 3 Results

### 3.1 Model performance and interpretability

**Fig 9:**
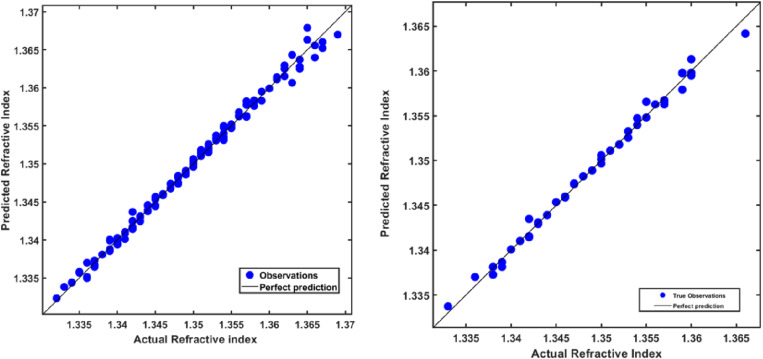
Scatter plots of predicted vs. experimental refractive index values for hemoglobin using the GPR model in training (left, R^2^: 99.4%) and testing datasets (right, R^2^:99.3%).

**Fig 10:**
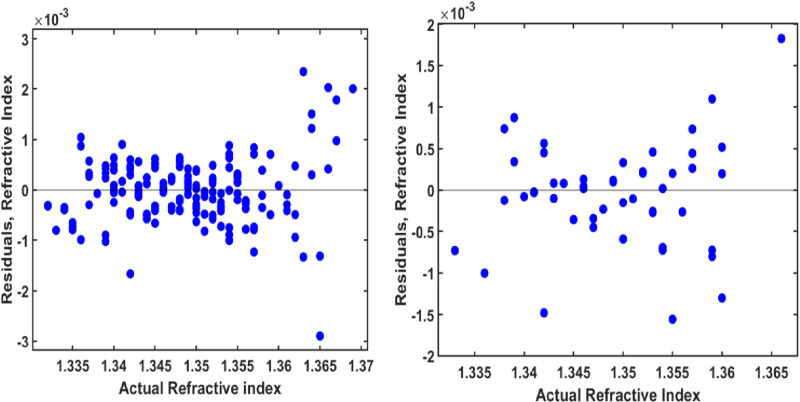
Residual plots for hemoglobin refractive index predictions using the GPR model in training (left) and testing datasets (right).

Fig 9 illustrates the relationship captured by the Gaussian Process Regression (GPR) model, demonstrating its ability to predict the refractive index of hemoglobin accurately. The model achieved high R^2^ values of 99.4% for the training set and 99.3% for the test set, as shown in Fig 9. Residual plots in Fig 10 reveal that the training dataset exhibits more significant variability in residuals, indicating some spread in the model’s predictions. In contrast, the testing dataset shows residuals tightly clustered around zero, suggesting strong generalization and accurate predictions on unseen data. This precision highlights the model’s capability to account for non-linear dependencies among hemoglobin concentration, wavelength, and refractive index, delivering reliable predictions across a broad spectrum.

Table 3 summarizes the performance metrics of the GPR model, underscoring its exceptional accuracy and reliability. The Root Mean Square Error (RMSE) values of 0.00061 for the training set and 0.00062 for the testing set reflect the model’s high precision. Similarly, the Mean Squared Error (MSE) values of 3.72 × 10 ⁻ ⁷ and 3.85 × 10 ⁻ ⁷ for the training and testing datasets indicate minimal discrepancies between predicted and actual refractive index values. The consistently high R^2^ values, exceeding 99% for both datasets, further validate the robustness of the GPR approach in modeling the complex optical properties of hemoglobin.

**Table 3:** Performance metrics for the GPR model on training and testing datasets, showing high accuracy with low error values and R^2^ above 99% for both datasets.

	Training Result			Testing Result				
Model Type	RMSE	MSE	R-Squared	MAE Testing	MSE Test	MAE Test	RMSE Test	R-Squared
GPR	0.00061	3.72E-07	99.403	0.000446	3.85E-07	0.000452	0.000621	99.337

The model’s predictive capabilities are particularly relevant for detecting and monitoring blood-related conditions. For example, in cases of anemia, which is characterized by low hemoglobin concentrations, the model can refine refractive index predictions to support early and accurate diagnosis. Similarly, in hemolysis, where hemoglobin concentration can vary rapidly, the model’s ability to provide real-time adjustments to diagnostic readings could improve diagnostic accuracy and clinical outcomes. By leveraging the GPR model’s precision, researchers and clinicians can gain deeper insights into hemoglobin’s optical behavior, paving the way for more effective diagnostic tools and improved patient care.

### 3.2 Validation results with external dataset

To evaluate the robustness and generalizability of the GPR model, an independent external dataset from Yahya and Saghir [34] was used for validation. This dataset comprises 35 refractive index measurements, with hemoglobin concentrations ranging from 0 to 260 g/L and wavelengths between 480 and 680 nm. The refractive index values in this dataset fall within the visible spectrum (1.3301 to 1.3879), similar to the training dataset. Importantly, the model had no prior exposure to this external dataset.

**Fig 11:**
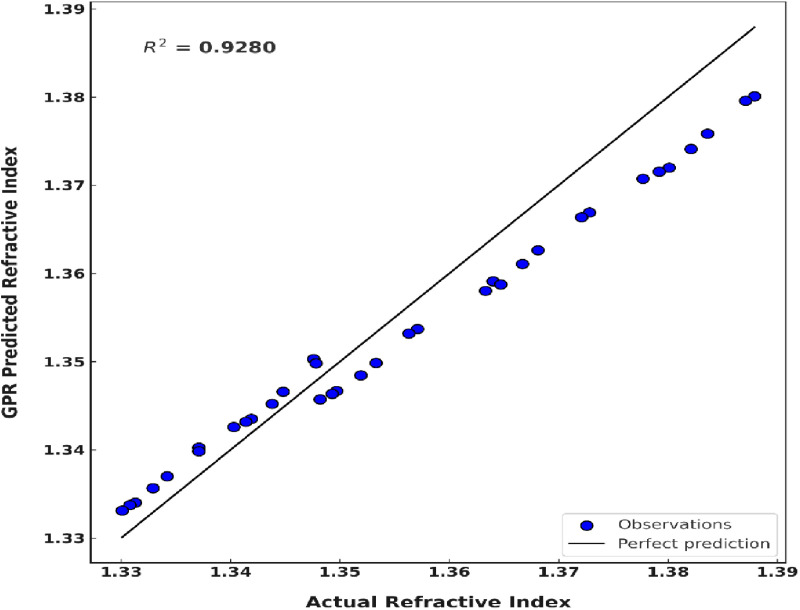
Comparison of actual refractive index values and GPR-predicted refractive index values, including the external validated dataset.

The GPR model demonstrated strong predictive performance, achieving an R^2^ value of 92.80%, RMSE of 0.0042, and MSE of 1.77 × 10 ⁻ ⁵. These results highlight the model’s ability to generalize across diverse datasets and accurately capture the relationships between wavelength, concentration, and refractive index. Fig 11 illustrates the comparison between the actual and GPR-predicted refractive index values, showing close alignment with the ideal line (y = x), which confirms the model’s precision.

It is important to mention that the slightly lower R^2^ value (92.80%) for the external dataset, compared to the training and testing sets, reflects the challenges of extrapolating to hemoglobin concentrations beyond 140 g/L. However, the low RMSE (0.0042) and MSE (1.77 × 10 ⁻ ⁵) indicate that the model’s absolute errors remain below 0.4%, which is clinically insignificant for optical diagnostics. Similar R^2^ reductions during external validation have been reported in comparable studies [43,44]. The model’s robust generalizability ensures its effectiveness when applied to new datasets, enhancing the accuracy and precision of optical diagnostics in real-world scenarios.

### 3.3 Interpretation through PDPs

**Fig 12:**
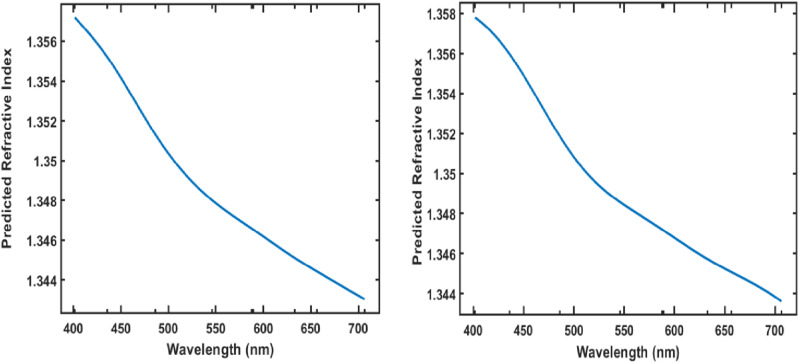
Partial Dependence Plots (PDPs) showing the effect of wavelength on the predicted refractive index of hemoglobin in the training dataset (left) and testing dataset (right).

The Partial Dependence Plot (PDP) analysis provides insights into how each input feature—wavelength and hemoglobin concentration—affects the refractive index predictions, enhancing the interpretability of the Gaussian Process Regression (GPR) model. The PDP for wavelength reveals a negative trend across both training and testing datasets, as illustrated in Fig 12. As wavelength increases, the predicted refractive index gradually decreases, aligning with known optical dispersion principles where longer wavelengths lead to lower refractive indices. This trend suggests that the model successfully captures the expected physical behaviour of hemoglobin’s optical properties, which is particularly relevant for biophotonic applications like optical coherence tomography (OCT), which rely on wavelength-specific responses. This inverse relationship, clearly visualized in the PDP, underscores the model’s ability to generalize accurately across the visible spectrum.

**Fig 13:**
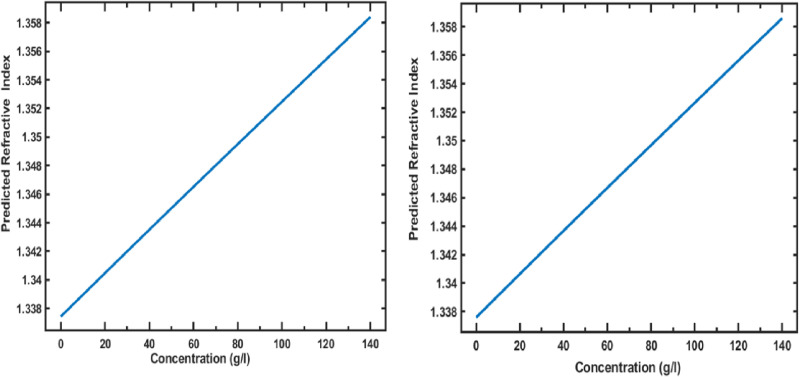
Partial Dependence Plots (PDPs) showing the effect of hemoglobin concentration on the predicted refractive index in the training dataset (left) and testing dataset (right).

In contrast, the PDP for hemoglobin concentration, shown in Fig 13 for the training and testing datasets, respectively, illustrates a positive trend. The refractive index increases with higher hemoglobin concentrations, reflecting hemoglobin’s denser optical characteristics and its effect on light propagation. This positive relationship reinforces the relevance of concentration as a critical factor in refractive index variations. By consistently capturing this dependency across both datasets, the model is robust in predicting how physiological variations in hemoglobin concentration influence its optical properties.

## 4. Discussion

The GPR model developed in this study demonstrates high predictive accuracy and interpretability, making it a valuable tool for advancing biophotonic diagnostics. The model achieved exceptional performance, with R^2^ values exceeding 99% for both training and testing datasets, and robust generalization on an independent external dataset (R^2^ = 92.80%, RMSE = 0.0042). These results underscore its potential for real-world applications, particularly in Optical Coherence Tomography (OCT) and reflectance spectroscopy, where precise refractive index predictions are critical.

A key strength of this study lies in the use of Partial Dependence Plots (PDPs), which enhance the model’s interpretability by visualizing the influence of wavelength and hemoglobin concentration on refractive index predictions. This transparency is especially valuable in clinical settings, where understanding the relationship between input features and predictions can build trust in the model and support its practical application.

The model’s ability to generalize across diverse datasets, including supraphysiological hemoglobin concentrations (up to 260 g/L), further highlights its robustness. However, the slight reduction in R^2^ during external validation (92.80%) points to challenges in extrapolating to extreme physiological conditions. While the absolute errors remain clinically insignificant (RMSE < 0.0042), this limitation suggests the need for future work to expand the model’s training data to include a broader range of concentrations and wavelengths, particularly in the near-infrared (NIR) spectrum. Extending the model to cover the NIR range, which is critical for deeper tissue imaging and non-invasive diagnostics, could significantly enhance its clinical utility.

Beyond hemoglobin, the GPR-based methodology presented here could be adapted to model the optical properties of other biological materials, such as collagen, melanin, or water. Such extensions could pave the way for a comprehensive suite of predictive models for medical imaging, enabling more precise diagnostics and treatment planning across a wide range of biomedical applications.

In conclusion, this study establishes GPR as a powerful and interpretable tool for predicting hemoglobin’s refractive index, with significant potential to improve the accuracy and reliability of optical diagnostic tools in clinical settings. Future research should focus on expanding the model’s applicability to the NIR spectrum and other biological materials, further advancing the field of biophotonic diagnostics.

## 5.0 Conclusion

This study demonstrates the effectiveness of Gaussian Process Regression (GPR) in accurately predicting the refractive index of hemoglobin across a wide range of wavelengths (400–700 nm) and concentrations (0–140 g/L). The GPR model achieved exceptional predictive accuracy, with R^2^ values of 99.4% for the training set and 99.3% for the testing set, along with low RMSE and MSE values, confirming its robustness. Validation using an independent external dataset further supported the model’s generalizability, with an R^2^ of 92.80%, RMSE of 0.0042, and MSE of 1.77 × 10 ⁻ ⁵. The integration of Partial Dependence Plots (PDPs) enhanced the model’s interpretability, providing clear insights into the influence of wavelength and concentration on refractive index predictions. While the model performs well within the tested range, its predictive accuracy decreases for hemoglobin concentrations beyond 140 g/L, as evidenced by the lower R^2^ value during external validation. This highlights the challenges of extrapolating the model to extreme physiological conditions. Future research should extend the model to include the near-infrared (NIR) spectrum, which is critical for deeper tissue imaging and non-invasive diagnostics. Additionally, the GPR-based approach could be adapted to model the optical properties of other biological materials, such as collagen and melanin, to further advance biophotonic diagnostics.

In summary, this study establishes GPR as a powerful tool for predicting hemoglobin’s refractive index, with significant potential to improve the accuracy and reliability of optical diagnostic tools like OCT and reflectance spectroscopy in clinical settings.
